# The Relationship Between Porphyromonas Gingivalis and Rheumatoid Arthritis: A Meta-Analysis

**DOI:** 10.3389/fcimb.2022.956417

**Published:** 2022-07-18

**Authors:** Yilin Li, Rui Guo, Patrick Kwabena Oduro, Tongke Sun, Hao Chen, Yating Yi, Weiqian Zeng, Qilong Wang, Ling Leng, Long Yang, Jun Zhang

**Affiliations:** ^1^ Department of Orthodontics, School and Hospital of Stomatology, Cheeloo College of Medicine, Shandong University, Jinan, China; ^2^ Shandong Key Laboratory of Oral Tissue Regeneration, Jinan, China; ^3^ Shandong Engineering Laboratory for Dental Materials and Oral Tissue Regeneration, Jinan, China; ^4^ Research center for Infectious Diseases, Tianjin University of Traditional Chinese Medicine, Tianjin, China; ^5^ School of Integrative Medicine, Tianjin University of Traditional Chinese Medicine, Tianjin, China; ^6^ Institute of Traditional Chinese Medicine, Tianjin University of Traditional Chinese Medicine, Tianjin, China; ^7^ State Key Laboratory of Component-Based Chinese Medicine, Ministry of Education, Tianjin, China

**Keywords:** porphyromonas gingivalis, rheumatoid arthritis, periodontitis, autoimmune disease, meta-analysis

## Abstract

Rheumatoid arthritis (RA) is a systematical autoimmune disease, characterized by chronic synovial joint inflammation and hurt. *Porphyromonas gingivalis*(*P. gingivalis*) can cause life-threatening inflammatory immune responses in humans when the host pathogenic clearance machinery is disordered. Some epidemiological studies have reported that *P. gingivalis* exposure would increase the prevalence of RA. However, the results remain inconsistent. Therefore, a meta-analysis was done to systematically analyze the relationship between *P. gingivalis* exposure and the prevalence of rheumatoid arthritis. Database including Cochrane Library, Web of Science, PubMed, and EMBASE were searched for published epidemiological articles assessed the relationship between *P. gingivalis* and RA. Obtained studies were screened based on the predefined inclusion and exclusion criteria. The overall Odds Ratios (ORs) of incorporated articles were pooled by random-effect model with STATA 15.1 software. The literature search returned a total of 2057 studies. After exclusion, 28 articles were included and analyzed. The pooled ORs showed a significant increase in the risk of RA in individuals with *P. gingivalis* exposure (OR = 1.86; 95% CI: 1.43-2.43). Subgroup analysis revealed that pooled ORs from populations located in Europe (OR = 2.17; 95% CI: 1.46-3.22) and North America (OR = 2.50; 95% CI: 1.23-5.08) were significantly higher than that from population in Asia (OR = 1.11; 95% CI: 1.03-1.20). Substantial heterogeneity was observed but did not significantly influence the overall outcome. In conclusion, our results indicated *P. gingivalis* exposure was a risk factor in RA. Prompt diagnosis and management decisions on *P. gingivalis* antimicrobial therapy would prevent rheumatoid arthritis development and progression.

## Introduction

Rheumatoid arthritis (RA) is a systemic autoimmune disease characterized by the production of anti-citrullinated protein antibodies (ACPA) (Kharlamova et al., 2016; Białowąs et al., 2020) and chronic synovial joint inflammation. If untreated or improperly controlled, this illness can lead to the destruction of cartilage and bone and decrease the quality of the patient’s life or even cause disability (Laugisch et al., 2016). RA affects about 1% of the population, with a female/male ratio of 2·5/1 (Bender et al., 2018). The disease can happen at any time in life, but its incidence increases with age, with individuals aged 40–70 years at an increased risk. Although what exactly causes RA remains unclear, several genetic alterations and environmental factors have been identified to contribute to RA pathogenesis (Eriksson et al., 2019).

Chronic periodontitis is the most common inflammatory disease worldwide, affecting 1/3 of the adult population. Both periodontal disease (PD) and RA display systemic markers of inflammation and share an association with HLA-DRB1 alleles and chronic inflammatory pathways (Äyräväinen et al., 2017). PD is much more frequent in RA patients compared with healthy people. Indeed, previous study reports have established a causal relationship between PD and RA (Scher and Abramson, 2013; Kim et al., 2018). Many clinical studies have indicated that patients with RA are more likely to exhibit periodontitis than those without RA (Berthelot and Le Goff, 2010). Individuals with RA also had higher levels of periodontal tissue destruction than the controls. Additionally, patients with periodontitis had a higher prevalence of RA than those without periodontitis.

Moreover, the elevated systemic inflammatory problem in people with PD has been coupled with an increased risk of chronic and potentially grievous diseases (Oluwagbemigun et al., 2019), including heart diseases, enterophthisis, kidney failure, cardiovascular diseases, diabetes, pulmonary diseases, premature infants, and cancers (Kononoff et al., 2020). In PD, the dysregulated immune inflammatory response is associated with the dysbiosis of the oral microbiota (Konig et al., 2015). *Porphyromonas gingivalis*(*P. gingivalis*) is a gram-negative, rod-shaped, obligate anaerobe of the oral cavity. *P. gingivalis* is the causative agent of the chronic inflammatory disease periodontitis. Strikingly, the association between RA and PD is because of the oral pathobiont *P. gingivalis* (Lee et al., 2015).

*P. gingivalis* and its infection are of clinical concern because of their numerous associations with potentially life-threatening diseases (Fisher et al., 2015). Previous studies report that *P. gingivalis* can induce an inflammatory response since some immunologic and inflammatory reactions are activated in the host, interfering with the bacterium’s clearance (Johansson et al., 2016). *P. gingivalis* can invade and penetrate different epithelial cells, and it has a complex mechanism that allows it to alter the cellular defense, notably some of the host’s unique genes (Schulz et al., 2019). Some studies have shown the effects of *P. gingivalis*, at diverse levels, on some molecules associated with cellular division. Gingival epithelial cells are the first natural barrier of the host periodontal tissue defense mechanism  (Moen et al., 2003; Okada et al., 2011; Mariette et al., 2020; Arévalo-Caro et al., 2022). *P. gingivalis* can adhere to gingival epithelial cells through specific adhesins, triggering various signaling pathways in gingival epithelial cells and causing its internalization in gingival epithelial cells to trigger cell dysfunction (Reichert et al., 2013).

Previous systematic reviews and clinical studies have linked *P. gingivalis* infection to RA (Bello-Gualtero et al., 2016). However, most of these studies were limited by small sample size, geographical location, and the absence of subgroup analysis (Leech and Bartold, 2015). In addition, unfortunately, findings on the link between *P.gingivalis* and RA remain heterogeneous, with some showing a positive correlation and others showing a null correlation between the two (Seror et al., 2015; Janssen et al., 2015). Therefore, current estimates of the risk of RA in *P. gingivalis* exposed individuals are needed to inform decisions on future drug and vaccine development (Ceccarelli et al., 2018). We, therefore, aimed to conduct a meta-analysis of the prevalence of *P. gingivalis* in patients with RA.

## Material and Methods

### Protocol and Registration

This meta-analysis was designed to investigate the relationship between *P. gingivalis* and RA, with the guidelines provided by the PRISMA statement (Liberati et al., 2009) and the Cochrane Handbook for Systematic Reviews of Interventions Version 5.1.0. Literature from the Cochrane Library, Web of Science, PubMed, and EMBASE were searched by April 14, 2022, with the search strategy of key words including rheumatoid arthritis and *P. gingivalis*. After conducting the literature search, the references of all returned studies were managed and screened.

### Eligibility Criteria

The searched articles were screened based the predefined eligible criteria: 1) epidemiological studies including cohort study, case-control study or cross-section study; 2) articles exploring the correlation between *P. gingivalis* and the risk of rheumatoid arthritis. When multiple research articles were available from the same population, the most recent peer-reviewed research article would be selected. On the contrary, articles would be excluded if they are: 1) inappropriate article types, such as comment, letters, review, meta-analysis; 2) results from animal models or *in vitro* experiments; 3) lack of detailed methodology to detect *P. gingivalis* exposure; 4) neither reporting exact odds ratio (OR) or risk ratio (RR), nor supplying enough data to calculate them; 5) articles with paradoxical data describing sample size in paragraphs or tables.

### Study Selection

The titles and abstracts from each database were downloaded and imported into the literature management software. Duplicate research articles were removed. Two independent reviewers (Yilin L and Rui G) screened the titles and abstracts in parallel based on the inclusion and exclusion criteria. Then the full text was retrieved and evaluated in depth. In addition, in scenarios where conflicts about a study could not be resolved after discussing the full-text among the two review authors, a third author (Tongke S) was consulted.

### Data Extraction

Two independent authors (Yilin L and Rui G) extracted data from included articles using a pre-piloted standardized data extraction form. The extracted data including author/year of publication, sample location (country), study design, sample size, match for control, biomarker used for determining *P. gingivalis* exposure and the reported or calculated OR and 95%CI.

### Quality Assessment

The quality of included articles were assessed by Agency for Healthcare Research and Quality (AHRQ) for cross-sectional studies and Newcastle-Ottawa Scale (NOS) for case-control and cohort studies (detailed items could be found in **Supplementary Materials Table 1** or in this website http://www.ohri.ca/programs/clinical_ epidemiology/oxford.asp). Yilin L and Rui G scored each item separately, and any discrepancies in the scores were resolved through discussion. All included studies were evaluated based on the final quality score, ranging from 0 to 10. Each study was assigned a low, moderate, or high quality based on the scores 0–5, 6–8, and 9–10, respectively.

### Statistical Analysis

Stata 15.1 software was used to conduct the meta-analysis. The relationship between *P. gingivalis* and RA was reported as odds ratio (OR) and 95% CI. Heterogeneity across studies was measured by I^2^ statistics. The fixed-effect model was used when the I^2^ was less than 50%, otherwise the random-effect model was used. Subgroup analysis was conducted to clarify the source of heterogeneity as well as to analyze the diversity among different subgroups. And sensitivity analysis were used to confirm whether the results were robust. Furthermore, Begg’s funnel plots and Egger’s test were used to examine publication bias across studies.

## Results

### Study Selection and Flow Diagram

**Figure 1:**
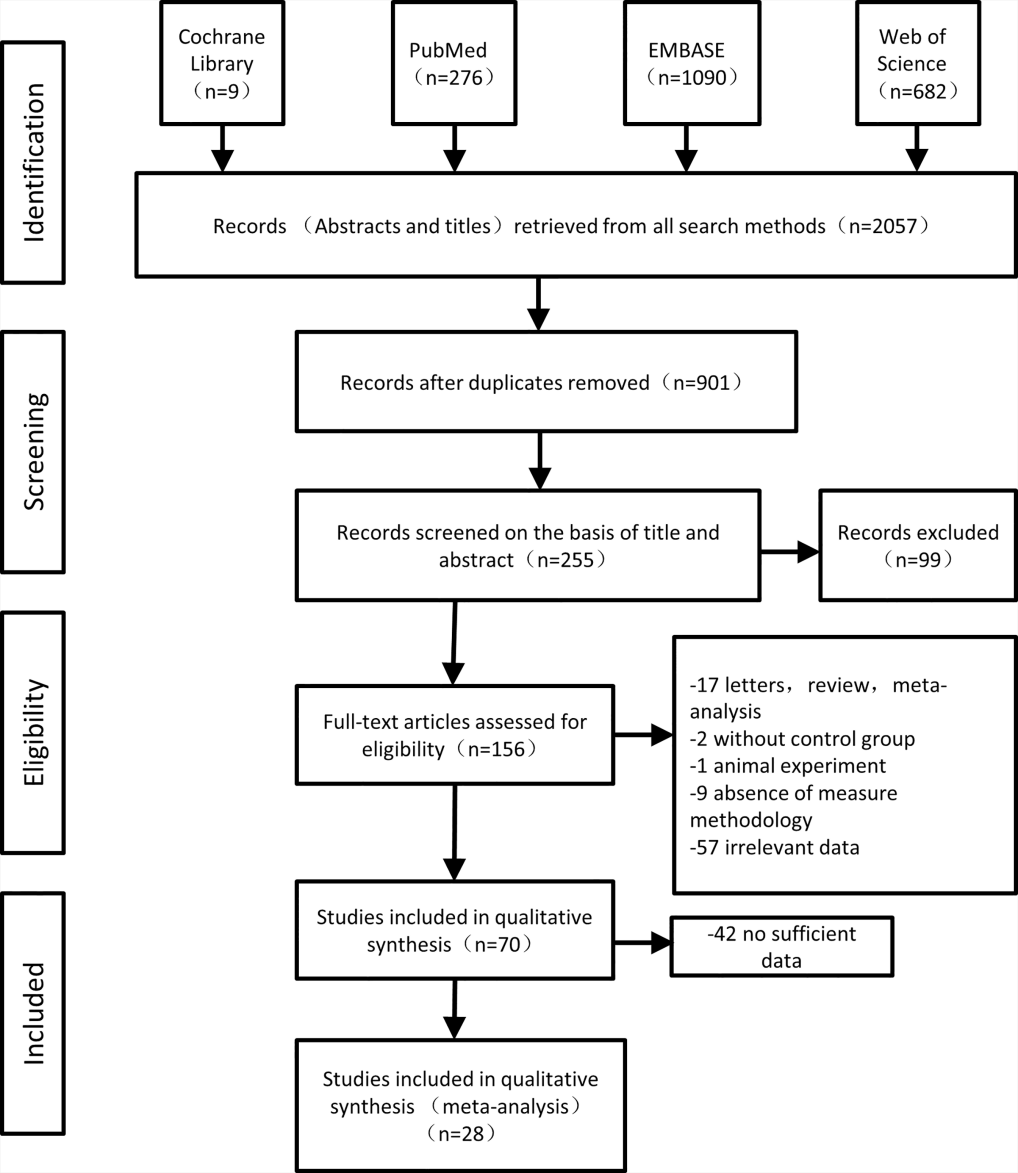
The flow diagram of the selection of the studies.

Our literature search identified 2057 studies. After removing duplicates, 901 records were screened for inclusion, among which 156 were proceeded for full-text eligibility and review. 86 studies were excluded based on inclusion and exclusion criteria, leaving 70 eligible studies for further analysis. Finally, after deeply evaluating 42 studies were excluded for insufficient data for OR or RR, yielding a total of 28 eligible studies for the synthesis (**Figure 1**). There were 2 articles reported two different ORs, those were OR calculated before RA treatment and OR after RA treatment (Äyräväinen et al., 2017; Martínez-Rivera et al., 2017). As most of these included articles reported ORs before treatment, in order to minimize the heterogeneity between studies, OR calculated before treatment was chosen in our meta-analysis.

### Characteristics of the Studies

Of the 28 studies, 16 were case-control studies, 4 were cohort studies, and 8 were cross-sectional studies, with a total of 4486 RA patients and 2607 controls. The included studies’ characteristics were displayed in **Table 1**. Among them, 17 study samples were in Europe, while 5 were in North America, 3 in Asia, and 3 in South America.

**Table 1:** Main characteristics of the studies selected for the systematic review and meta-analysis.

Study	Country	Sample location	Study design	Sample size		Matched	OR (95%CI)	Biomarker	NOS score
				RA	Control				
**Arévalo-Caro et al., 2022**	Columbia	South America	cross-sectional	50	50	age&sex	0.61 (0.27, 1.36)	anti-*P. gingivalis*	8
**Arvikar et al., 2013**	USA	North America	cohort study	93	72	age	4.42 (1.81, 10.80)	anti-*P. gingivalis*	6
**Äyräväinen et al., 2017**	Finland	Europe	cohort study	71	27	age&sex&community	2.29 (0.47, 11.10)	*P. gingivalis*	9
**Bello-Gualtero et al., 2016**	Columbia	South America	cross-sectional	48	48	age&sex	0.54 (0.24, 1.24)	*P. gingivalis*	8
**Bender et al., 2018**	Switzerland	Europe	case-control	10	10	age&ethnicity	9.80 (0.44, 219.25)	*P. gingivalis*	8
**Ceccarelli et al., 2018**	Italy	Europe	case-control	143	57	NA	1.92 (1.01, 3.64)	*P. gingivalis*	6
**Fisher et al., 2015**	Espana	Europe	case-control	103	309	age&sex	0.66 (0.17, 2.62)	anti-RgpB	8
**Janssen et al., 2015**	Netherlands	Europe	case-control	86	36	NA	14.60 (0.85, 251.68)	*P. gingivalis*	5
**Jasemi et al., 2021**	Italy	Europe	case-control	148	148	age&sex	3.08 (1.58, 5.99)	anti-RgpA	8
**Johansson et al., 2016**	Sweden	Europe	case-control	192	198	age&sex	1.20 (0.75,1.92)	anti-RgpB	9
**Kharlamova et al., 2016**	Sweden	Europe	case-control	1974	377	age&sex&residential area	2.96 (2.00, 4.37)	anti-RgpB	9
**Kim et al., 2018**	Korea	Asia	cross-sectional	260	86	age&sex	0.33 (0.04, 2.63)	*P. gingivalis*	7
**Kirchner et al., 2017**	Germany	Europe	cross-sectional	103	104	age	1.10 (0.64,1.90)	*P. gingivalis*	7
**Kononoff et al., 2020**	Finland	Europe	case-control	53	82	age	2.55 (1.25, 5.22)	anti-*P. gingivalis*	6
**Laugisch et al., 2016**	Switzerland	Europe	case-control	52	44	age	2.05 (0.89, 4.73)	*P. gingivalis*	8
**Maldonado et al., 2020**	Switzerland	Europe	case-control	26	72	NA	1.27 (0.51, 3.14)	*P. gingivalis*	7
**Martínez-Rivera et al., 2017**	Mexico	North America	cross-sectional	132	10	NA	5.30 (1.80, 6.10)	*P. gingivalis*	6
**Mikuls et al., 2009**	USA	North America	case-control	78	40	age&sex	3.00 (1.36, 6.60)	anti-*P. gingivalis*	8
**Mikuls et al., 2012**	USA	North America	cohort study	113	171	NA	1.41 (1.08, 1.85)	anti-*P. gingivalis*	6
**Okada et al., 2011**	Japan	Asia	case-control	80	38	age&sex&smoking	1.11 (1.03, 1.20)	anti-*P. gingivalis*	8
**Rahajoe et al., 2021**	Indonesia	Asia	cohort study	70	70	age&sex&smoking&number	1.20 (0.52, 2.74)	*P. gingivalis*	9
**Reichert et al., 2013**	Germany	Europe	case-control	42	114	age	5.50 (1.50, 19.90)	*P. gingivalis*	8
**Rinaudo-Gaujous et al., 2019**	France	Europe	case-control	79	27	NA	1001.00 (87.14, 11499.17)	anti-*P. gingivalis*	5
**Rodríguez et al., 2021**	Columbia	South America	cross-sectional	51	51	age&sex	4.43 (1.86, 10.52)	*P. gingivalis*	8
**Scher et al., 2012**	USA	North America	case-control	65	18	age&sex&ethnicity	0.73 (0.18, 2.88)	*P. gingivalis*	7
**Schmickler et al., 2017**	Germany	Europe	cross-sectional	168	168	age&sex&smoking	1.05 (0.68, 1.61)	*P. gingivalis*	7
**Schulz et al., 2019**	Germany	Europe	case-control	101	100	sex	4.67 (2.07, 10.50)	*P. gingivalis*	7
**de Smit et al., 2014**	Netherlands	Europe	cross-sectional	95	80	age&sex	0.75 (0.34, 1.63)	*P. gingivalis*	8

NA, not available; not mentioned.

### Data Synthase

**Figure 2:**
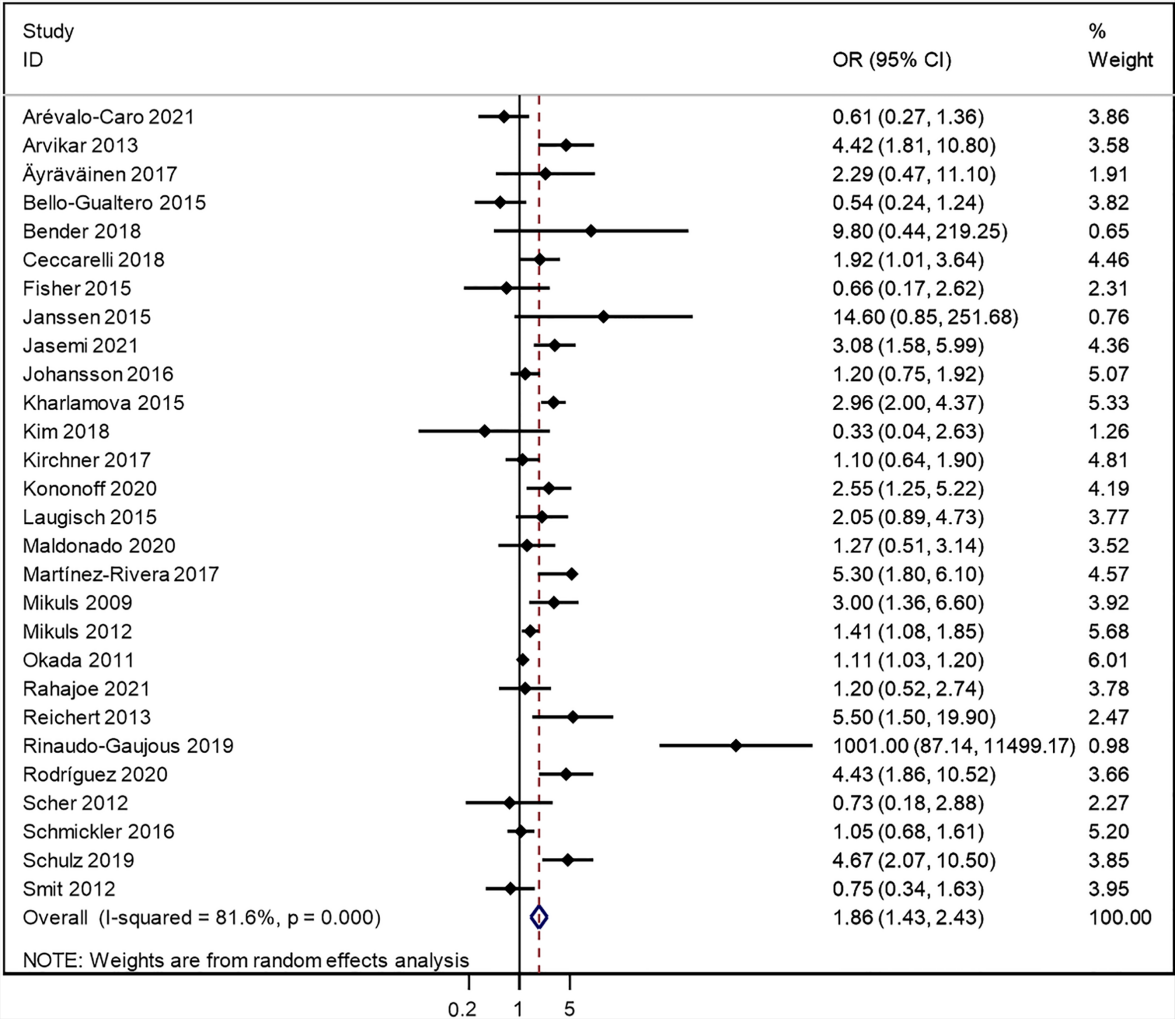
Forest plot for the association between porphyromonas gingivalis (*P. gingivalis*) and rheumatoid arthritis (RA) of dichotomous data.

These 28 studies represented 28 independent populations. Results from our meta-analysis showed a significant increase in the risk of rheumatoid arthritis in individuals with *P. gingivalis* exposure, with the overall pooled OR 1.86 (95%CI: 1.43, 2.43, **Figure 2**). There was apparent heterogeneity between these included articles, as the I^2^ was 81.6% and the estimate of between-study variance Tau-squared was 0.3015. Therefore, the random-effect model was used. Results from Begg’s funnel plot and Egger’s tests indicated that there might exist some publish bias (Egger’s text p= 0.004) (**Supplementary Figure 1**).

As in Arévalo-Caro’s study (Arévalo-Caro et al., 2022), two different ORs were reported because different biomarkers (IgG1 anti-*P. gingivalis* and IgG2 anti-*P. gingivalis*) were used to define *P. gingivalis* exposure. To compare these two different biomarkers on the pooled ORs, the two ORs were calculated separately. The pooled OR showed that *P. gingivalis* exposure defined by IgG1 anti-*P. gingivalis* or IgG2 anti-*P. gingivalis* had limited effect on the final results of the meta-analysis, as the pooled OR were 1.86 vs 1.90 **(** **Supplementary Figure 2** **)** in IgG1 anti-*P. gingivalis* and IgG2 anti-*P. gingivalis* respectively. In our pooled data, the OR from IgG1 anti-*P. gingivalis* were mainly chosen.

### Subgroup Analysis

**Figure 3:**
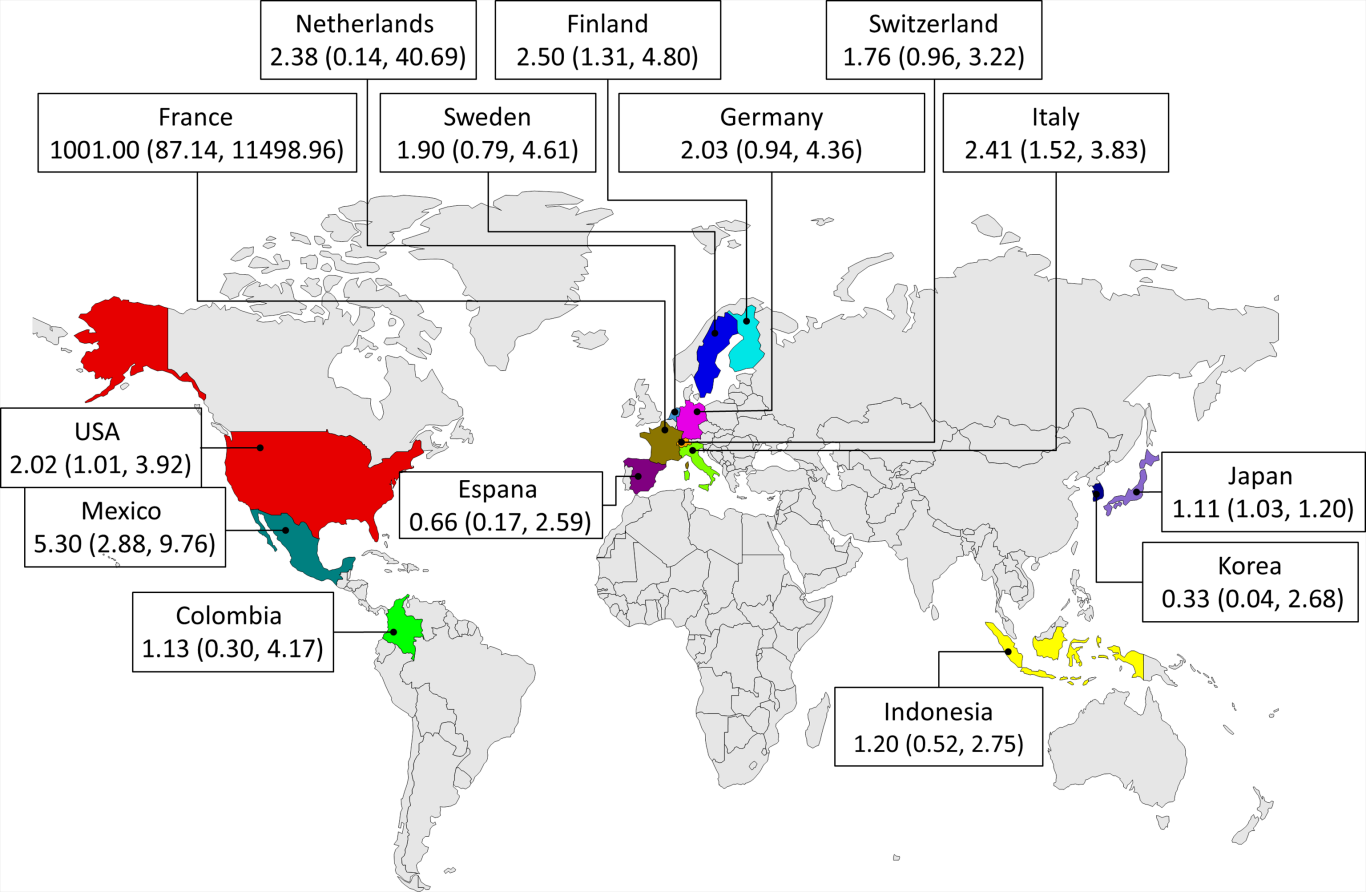
Subgroup analyses of the association between *P. gingivalis* and rheumatoid arthritis in different countries.

To determine the primary sources of heterogeneity, we conducted a subgroup analysis based on study design (case-control, cohort or cross-sectional study), OR’s sources (reported by the authors or calculated based on their original data), sample size (RA patients ≥150 or < 150), population locations (countries), the biomarker of *P. gingivalis* (anti-*P. gingivalis*, *P. gingivalis* anti-RgpB or anti-RgpA), the detection methods in evaluation *P. gingivalis* exposure (ELISA, PCR or bacterial culture), the quality (NOS ≥ 8 or < 8) and the population location (continent) (**Figure 3** and **Table 2**). Unfortunately, none of these factors could well explain heterogeneity between studies. Instead, we found that the association might be stronger in Europe and North America than Asia, as the pooled ORs from populations located in Europe (OR = 2.17; 95% CI: 1.46-3.22) and North America (OR = 2.50; 95% CI: 1.23-5.08) were significantly higher than that from population in Asia (OR = 1.11; 95% CI: 1.03-1.20).

**Table 2:** Results of subgroups analysis.

subgroup analysis types	Subgroups	No. of studies involved	Meta-OR (95% CI)	Heterogeneity between studies (I^2^)
**Study design**	Case-control	16	2.42 (1.60, 3.67)	84.4%
	Cohort	4	1.83 (1.05, 3.19)	52.8%
	Cross-sectional	8	1.22 (0.66, 2.26)	82.8%
**Sample size**	Cases ≥150	4	1.38 (0.71, 2.68)	82.2%
	Cases < 150	24	1.99 (1.47, 2.70)	81.6%
**ORs sources**	Reported by authors	8	2.09 (1.36, 3.21)	89.3%
	Calculated based on original data	20	1.82 (1.22, 2.70)	74.5%
**Biomarker of *P. gingivalis***	*P. gingivalis*	17	1.76 (1.18, 2.64)	70.7%
	anti-*P.gingivalis*	7	2.14 (1.28, 3.60)	88.9%
	anti-RgpB	3	1.54 (0.69, 3.47)	81.6%
	anti-RgpA	1	3.08 (1.58, 6.00)	/
**Detection methods**	ELISA	11	2.02 (1.36, 3.01)	88.1%
	PCR	10	1.99 (1.19, 3.32)	79.3%
	Bacterial culture	7	1.23 (0.70, 2.17)	26.7%
**Quality**	NOS ≥ 8	15	1.61 (1.14, 2.28)	78%
	NOS < 8	13	2.32 (1.44, 3.75)	82%
**Sample location (continent)**	South America	3	1.13 (0.30, 4.17)	86.5%
	North America	5	2.50 (1.23, 5.08)	81.9%
	Europe	17	2.17 (1.46, 3.22)	75.9%
	Asia	3	1.11 (1.03, 1.20)	0.0%

### Sensitivity Analysis

**Figure 4:**
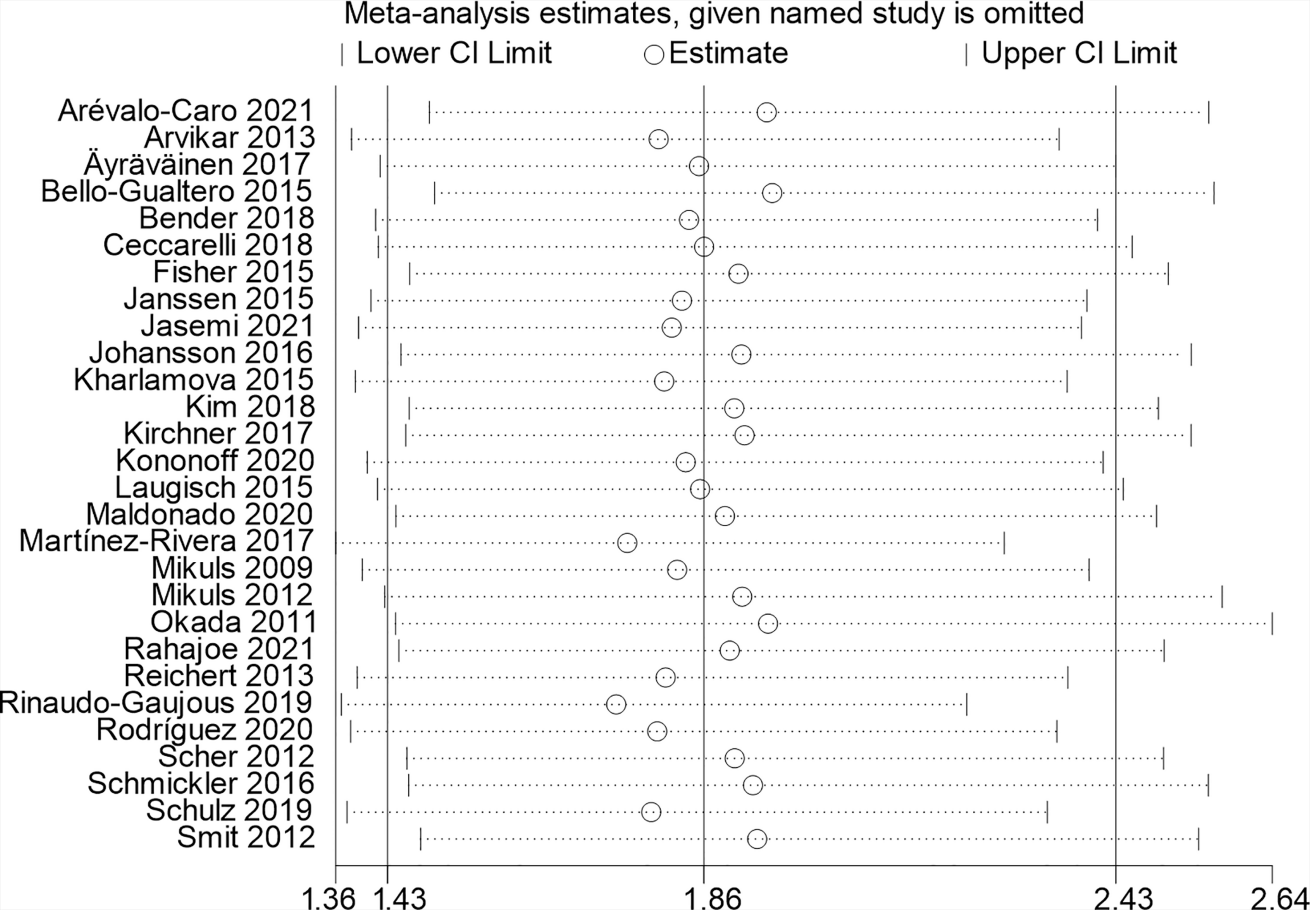
Sensitivity analysis.

A sensitivity analysis (**Figure 4**) was performed after excluding one of the 28 included studies to evaluate the impact of an individual study on the overall outcome. The overall sensitivity analyses indicated that our results were robust, omitting anyone of the studies would not significantly interfere with the overall measured outcome.

## Discussion

The relationship between *P. gingivalis* and RA remains heterogeneous and a subject of concern (Chaparro-Sanabria et al., 2019). Our meta-analysis showed that a patients exposed to *P. gingivalis* would have a higher risk of RA. According to Seror et al.’s study, there was a significant difference between the early RA group and the HC group for the frequency of *P. gingivalis*, which is the same as Goh et al.’s study (Seror et al., 2015; Goh et al., 2016). Contrary to our results, Seror et al.’s and Gon et al.’s studies revealed that they did not detect any association of anti-*P. gingivalis* antibodies with RA or ACPA status (Kharlamova et al., 2017). These results suggest that the association of periodontitis and RA could be linked to bacterial species other than *P. gingivalis* or a mechanism other than citrullination (**Figure 4**). This disparity might be due to various factors, including patient health, sample size, and RA treatment types (Kirchner et al., 2017).

Another study by Schmickler and others showed that patients with RA had worse oral health than healthy controls because of the absolute number of missing teeth and periodontal conditions (Rahajoe et al., 2021). The prevalence of all bacteria investigated in RA and HC groups is similar (Arévalo-Caro et al., 2022). The course of the disease, drug for rheumatism, and restricted motor skills, on the other hand, did not affect periodontal conditions (Janssen et al., 2015), even though there was a statistically significant association between a rheumatoid factor and periodontal condition (Kim et al., 2018). Their studies also reported that within the RA group, higher serum levels of *P. gingivalis* may be a significant marker for the higher burden of the bacteria and invasive periodontal diseases (Martínez-Rivera et al., 2017), which may justify why this bacterium is the only periodontal pathogen correlated with a higher risk of RA (Kononoff et al., 2021).

Also, a comparison between saliva samples of healthy individuals and patients with RA in a recent study indicated a significant relationship between the higher prevalence of *P. gingivalis* and RA (Mikuls et al., 2009; Schmickler et al., 2017). The possible relationship between RA and *P. gingivalis* in different studies has been investigated, and most of these studies have shown a positive relationship between them (Unriza-Puin et al., 2017). A study from the USA reported a positive relationship between *P. gingivalis* and periodontal diseases (Mikuls et al., 2012). Also, it revealed that *P. gingivalis* was the most potent predictor of periodontal diseases among adolescents (Janssen et al., 2015; Manoil et al., 2021). The importance of our findings of a higher correlation rate of *P. gingivalis* in RA compared to healthy tissue is uncertain (Rodriguez et al., 2021). However, this bacterium invades healthy and inflammatory tissue more than other bacteria such as candida (de Smit et al., 2014; Rinaudo-Gaujous et al., 2019). Meanwhile, *P. gingivalis* can intervene in the inflammatory process by inhibiting the host’s cellar death and inducing cell proliferation (Arvikar et al., 2013).

It is suggested that anti-*P. gingivalis* antibody levels play a significant role in the pathogenesis of RA (Westra et al., 2021), while distinct similarities are shown in both RA and periodontitis (Papageorgiou et al., 2020). The data suggest that RA and periodontitis may be linked to the periodontal pathogen *P. gingivalis* (Maldonado et al., 2020). Citrullinated variants of the Fibrin chains are considered target autoantigens in the rheumatoid joint (Perricone et al., 2019). *P. gingivalis* is the only prokaryotic organism that produces PPAD and contributes to the initiation of ACPA generation because of PPAD expression (Quirke et al., 2014). For example, PAD expressed in the joint deaminates synovial fibrin (Valor and de la Torre Ortega, 2012), and these synovial antigens may serve as targets for auto-antibody formation triggered by *P. gingivalis* associated with periodontitis and epitope spreading (Lee et al., 2015). Because antibody against citrullinated proteins plays a significant role in autoimmunity in RA (Xiao et al., 2020; Jasemi et al., 2021), an assumption is made that individuals predisposed to periodontitis are exposed to citrullinated antigens that become systemic immunogens (Moen et al., 2003). A study shows that antibody responses to *P. gingivalis* may affect ACPA responses, and the data support a role for the oral pathogen *P. gingivalis* in the etiology of RA (Arévalo-Caro et al., 2022).

There are differences between the prevalence rates reported by different studies for *P. gingivalis* that may be associated with the variations of *P. gingivalis* strains used in different studies (Ziebolz et al., 2011; Scher et al., 2012). Two completely different *P. gingivalis* strains differ in several aspects. For instance, the strain *P. gingivalis* ATCC53978 has a recognized capsule as the primary antigen associated with pathogenesis, but the strain *P. gingivalis* ATCC33277 does not have this antigen. Therefore, it leads to inflammation slightly (Sayehmiri et al., 2015). This meta-analysis did not have enough age, gender, smoking, or alcohol consumption data. These variables are essential and may influence the ability of bacteria to invade the immune system during the invasion of tissues and, therefore, potentially influence the inflammatory process.

As we know, sample size and location were critical factors in meta-analysis. Thus in our subgroup analysis, these included studies were distributed to two groups based on sample size (indicated by RA cases ≥150 or < 150), and ORs were pooled respectively. Our results showed that the differences between these two groups were statistically insignificant, as there was an overlap in 95%CI of individual ORs. In addition, we further analyzed pooled ORs in different sample locations in two ways. Firstly, the included studies were distributed to four groups based on their continents. Then the included studies were distributed to fourteen groups based on their countries. Our results showed there exists some regional differences in the relationship between P. gingivalis exposure and RA, as the pooled ORs from populations located in Europe and North America were significantly higher than that located in Asia. Also, pooled ORs from population located in Italy, Finland and Mexico were relatively higher than that in Japan (**Figure 3**). This might be due to the lower prevalence of RA in Japan (Silman and Pearson, 2002). It was a pity that no study reported any information about the relationship between P. gingivalis exposure and RA from population located in China. More studies are warranted to clarify the relationship in Chinese populations. Overall, our results suggested that it should be noted that P. gingivalis was a risk factor in RA development in populations around the world, even though the ORs were different across different areas.

This paper mainly conducted a meta-analysis on different regions, populations, research methods, age, sample size and other factors. The results indicated *P. gingivalis* exposure was a risk factor in RA. Prompt diagnosis and management decisions on *P. gingivalis* antimicrobial therapy would prevent rheumatoid arthritis development and progression. But, factors such as people smoked or not, drank alcohol, congenital hypoplasia, and whether individual has underlying medical conditions were not taken into account. Furthermore, in the included studies, different exposure methods were adopted for different biomarkers. Enzyme-Linked Immunosorbent Assay (ELISA) is generally used for the detection of serum antibodies, (e.g. Arvikar et al., 2013). There were two ways to detect *P. gingivalis*: one was bacterial culture (e.g. Janssen et al., 2015) and the other was Polymerase Chain Reaction (PCR) (e.g. Reichert et al., 2013). Results from subgroup analysis showed that the pooled OR were slightly higher in ELISA or PCR groups compared with Bacterial culture group, suggested that different detection method might influence the association between *P. gingivalis* and RA. This may provide a direction for future research. Meanwhile, a range of biomarkers were involved in the original text, including *P. gingivalis*, anti-*P. gingivalis*, anti-RgpB and anti-RgpA. The impact of different biomarkers was also evaluated in our subgroup analysis. The results showed that the differences were not that obvious. More studies are warranted to compare these difference directly.

As a chronic autoimmune disease, Rheumatoid arthritis is developed due to genetics and environmental risks, and periodontitis is one of the consistently reported risk factors (González-Febles and Sanz, 2021). Most importantly, *P. gingivalis* is one of the commonly found bacterium in periodontitis patients (Jia et al, 2019). It was reported that *P. gingivalis* has a unique ability to produce citrullinate proteins or peptides by proteolytic cleavage at Arg-X peptide bonds by arginine gingipains, followed by citrullination of carboxy-terminal arginines by bacterial PAD (Smolen et al., 2016). Specific citrullinated peptides generated by P. gingivalis could be worked as self-antigen and lead to the breakdown of immune tolerance at the site of gingival inflammation (Muñoz-Atienza et al, 2020). Epitope spreading to other host citrullinated proteins would lead to chronic and destructive inflammation in the joint, which triggers the development of rheumatoid arthritis (Wegner et al, 2010).

In conclusion, our results indicated P. gingivalis exposure was a potential risk factor in RA. More perspective studies and mechanism research are warranted to confirm the causal link between *P. gingivalis* exposure and RA process. Prompt diagnosis and management decisions on *P. gingivalis* antimicrobial therapy would prevent RA development and progression.

## Author Contributions

JZ and LY conceived the project. YL, RG and LL designed the whole meta-analysis, including article search and exclusion, quality assessment, and analyzed the data. LL, JZ and LY supervised the project, provided the funding, interpreted the results. YL and RG wrote the manuscript with input from all authors. PO helped revise languages. TS, HC, YY and WZ helped with the data analysis. QW reviewed the data, provided advice and funding. All authors contributed to the article and approved the submitted version.

## Funding

This study was supported by the National Key Research and Development Program of China (2019YFC1708803), the Science and Technology Program of Tianjin, China (21ZYJDJC00070), Innovation Team and Talents Cultivation Program of National Administration of Traditional Chinese Medicine (ZYYCXTD-C-202203) and the Education Committee of Tianjin (2021KJ130).

## Conflict of Interest

The authors declare that the research was conducted in the absence of any commercial or financial relationships that could be construed as a potential conflict of interest.

## Publisher’s Note

All claims expressed in this article are solely those of the authors and do not necessarily represent those of their affiliated organizations, or those of the publisher, the editors and the reviewers. Any product that may be evaluated in this article, or claim that may be made by its manufacturer, is not guaranteed or endorsed by the publisher.

## Supplementary Material

Supplementary Figure 1 Publication bias in the risk difference (RD) in dichotomous data, Begg’s funnel plot.

Supplementary Figure 2 Two different ORs were reported for **(A)** IgG1 anti-*P. gingivalis*, **(B)** IgG2 anti-*P. gingivalis* were used to define *P. gingivalis* exposure.
