# Supplementary figures and images for: The Relationship Between Porphyromonas Gingivalis and Rheumatoid Arthritis: A Meta-Analysis
Source: Front Cell Infect Microbiol. 2022 Jul 18;12:956417. doi: 10.3389/fcimb.2022.956417 (PMC9340274; doi:10.3389/fcimb.2022.956417)

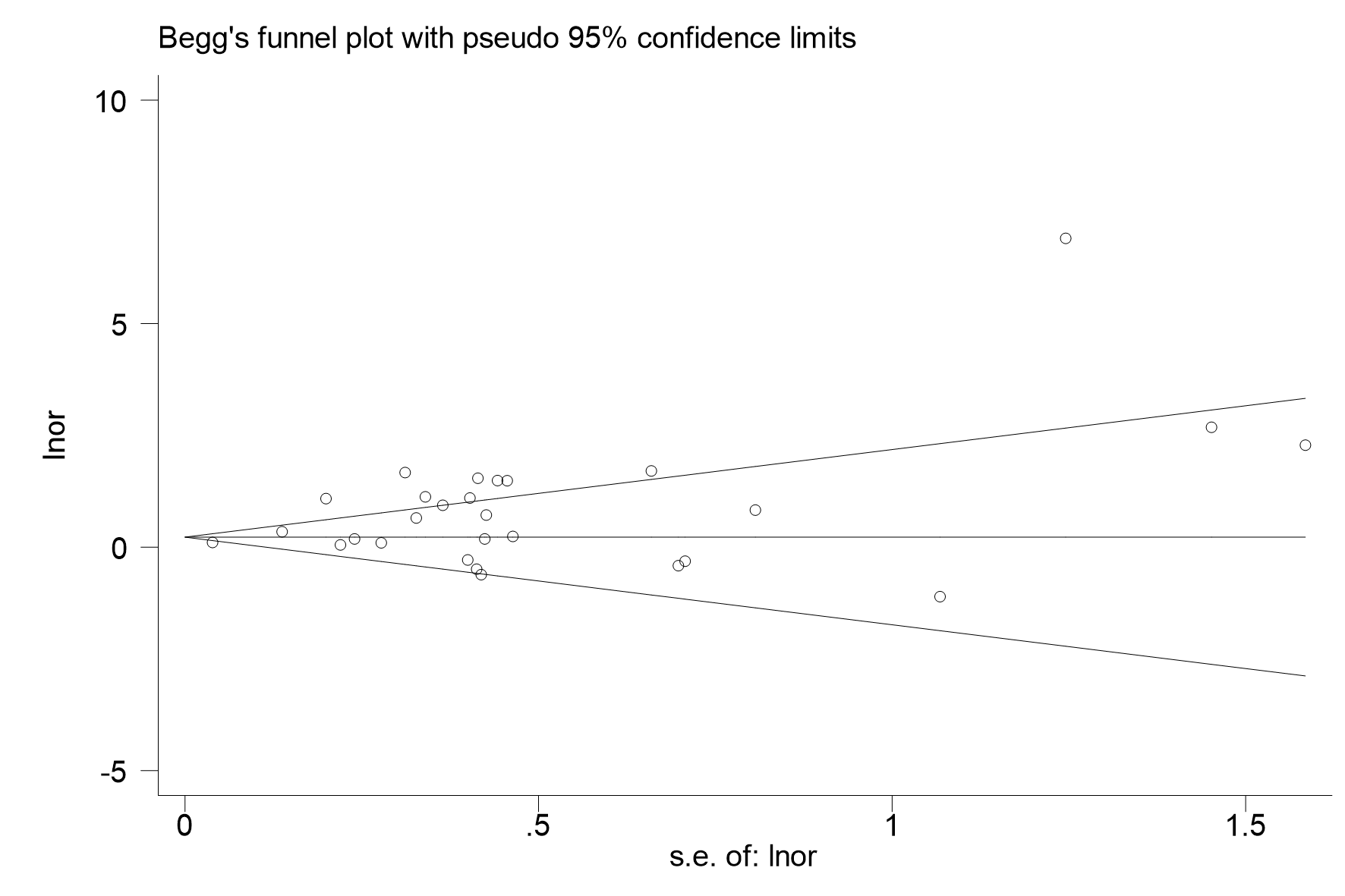

Supplement: Supplementary Figure 1 — Publication bias in the risk difference (RD) in dichotomous data, Begg’s funnel plot. [file Image_1.tif]

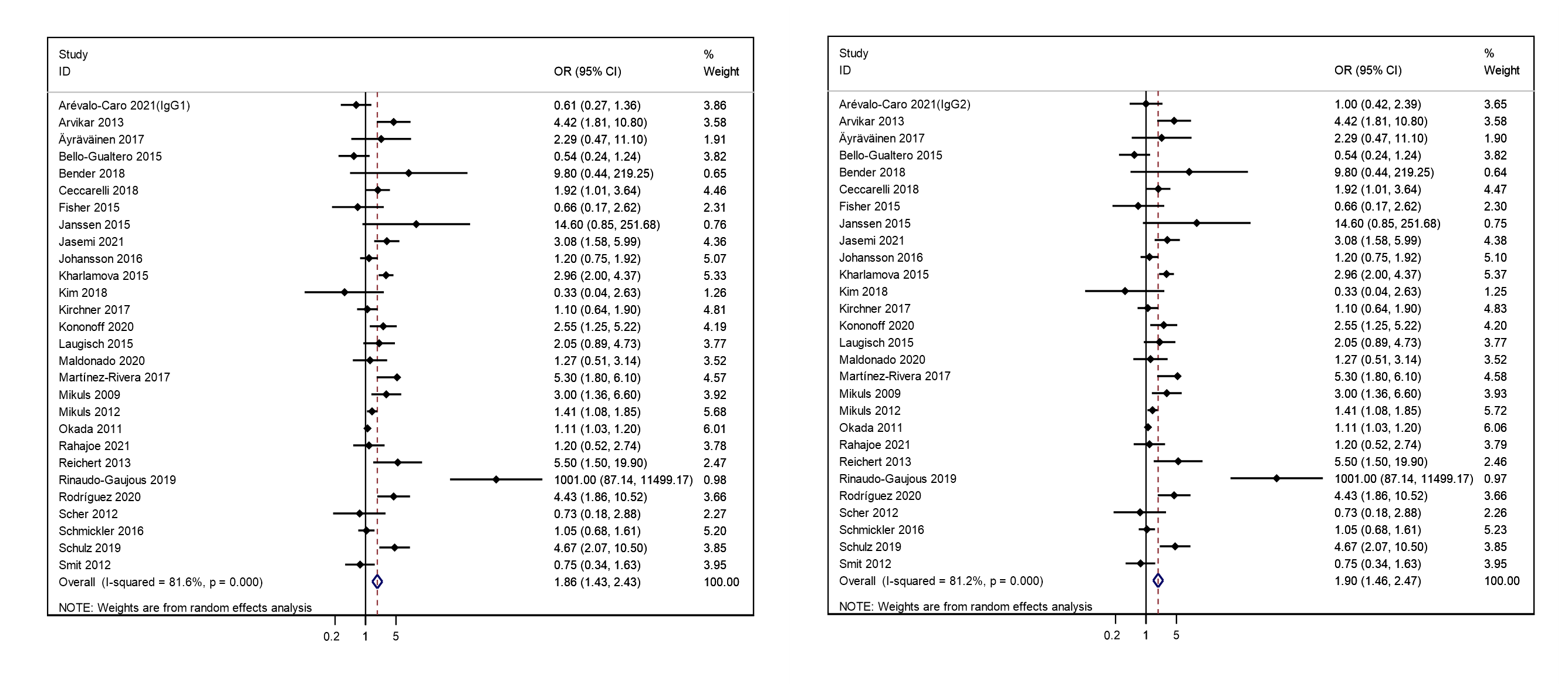

Supplement: Supplementary Figure 2 — Two different ORs were reported for (A) IgG1 anti-P. gingivalis, (B) IgG2 anti-P. gingivalis were used to define P. gingivalis exposure. [file Image_2.tif]
