# Supplementary material for: The Relationship Between Porphyromonas Gingivalis and Rheumatoid Arthritis: A Meta-Analysis
Source: Front Cell Infect Microbiol. 2022 Jul 18;12:956417. doi: 10.3389/fcimb.2022.956417 (PMC9340274; doi:10.3389/fcimb.2022.956417)
Supplement: Supplementary file 3 [file Table_1.docx]

**Table S1: Newcastle - Ottawa Quality Assessment Scale**

| Study design |  | Items | Answer | Score |
| --- | --- | --- | --- | --- |
| Case control study | Selection | The case definition is adequate or not | Yes, with independent validation | 1 |
|  |  |  | Yes, eg record linkage or based on self-reports | 0 |
|  |  |  | No description | 0 |
|  |  | Representativeness of the cases | Consecutive or obviously representative series of cases | 1 |
|  |  |  | Potential for selection biases or not stated | 0 |
|  |  | Selection of Controls | Community controls | 1 |
|  |  |  | Hospital controls | 0 |
|  |  |  | No description | 0 |
|  |  | Definition of Controls | No history of disease (endpoint) | 1 |
|  |  |  | No description of source | 0 |
|  | Comparability | Comparability of cases and controls on the basis of the design or analysis | Study controls for __ (Select the most important factor.) | 1 |
|  |  |  | Study controls for any additional factor | 1 |
|  | Exposure | Ascertainment of exposure | Secure record (eg surgical records) | 1 |
|  |  |  | Structured interview where blind to case/control status | 1 |
|  |  |  | Interview not blinded to case/control status | 0 |
|  |  |  | Written self-report or medical record only | 0 |
|  |  |  | No description | 0 |
|  |  | Same method of ascertainment for cases and controls | Yes | 1 |
|  |  |  | No | 0 |
|  |  | Non-Response rate | Same rate for both groups | 1 |
|  |  |  | Non respondents described | 0 |
|  |  |  | Rate different and no designation | 0 |
| Cohort study | Selection | Representativeness of the exposed cohort | Truly representative of the average ______ (describe) in the community | 1 |
|  |  |  | Somewhat representative of the average ____ in the community | 1 |
|  |  |  | Selected group of users eg nurses, volunteers | 0 |
|  |  |  | No description of the derivation of the cohort | 0 |
|  |  | Selection of the non-exposed cohort | From the same community | 1 |
|  |  |  | From a different source | 0 |
|  |  |  | No description | 0 |
|  |  | Ascertainment of exposure | Secure record (eg surgical records) | 1 |
|  |  |  | Structured interview | 1 |
|  |  |  | Written self-report | 0 |
|  |  |  | No description | 0 |
|  |  | Demonstration that outcome of interest was not present at start of study | Yes | 1 |
|  |  |  | No | 0 |
|  | Comparability | Comparability of cohorts on the basis of the design or analysis | study controls for _____ (select the most important factor) | 1 |
|  |  |  | study controls for any additional factor | 1 |
|  | Outcome | Assessment of outcome | Independent blind assessment | 1 |
|  |  |  | Record linkage | 1 |
|  |  |  | Self-report | 0 |
|  |  |  | No description | 0 |
|  |  | Follow-up long enough for outcomes to occur | Yes | 1 |
|  |  |  | No | 0 |
|  |  | Adequacy of follow up of cohorts | Complete follow up - all subjects accounted for | 1 |
|  |  |  | Subjects lost to follow up unlikely to introduce bias - small number lost - > ____ % (select an adequate %) follow up, or description provided of those lost) | 1 |
|  |  |  | Follow up rate < ____% (select an adequate %) and no description of those lost | 0 |
|  |  |  | No statement | 0 |

Note:

1, A study can be awarded a maximum of one star for each numbered item within the Selection and Exposure categories. A maximum of two stars can be given for Comparability in case control studies.

2, A study can be awarded a maximum of one star for each numbered item within the Selection and Outcome categories. A maximum of two stars can be given for Comparability in cohort studies.

**Appendix: Information of 156 articles**

| No. | DOI | Title |
| --- | --- | --- |
| 1 | 10.1111/jre.12443 | Active Matrix Metalloproteinase-8 And Periodontal Bacteria Depending On Periodontal Status In Patients With Rheumatoid Arthritis |
| 2 | 10.1007/s00784-020-03469-0 | Adipokines And Periodontal Markers As Risk Indicators Of Early Rheumatoid Arthritis: A Cross-Sectional Study |
| 3 | 10.1016/j.sjbs.2020.04.040 | Analysis Of Subgingival Microbiome Of Periodontal Disease And Rheumatoid Arthritis In Chinese: A Case -Control Study |
| 4 | 10.1136/annrheumdis-2013-204594 | Antibodies Against Porphyromonas Gingivalis In Seropositive Arthralgia Patients Do Not Predict Development Of Rheumatoid Arthritis |
| 5 | 10.1002/art.23936 | Antibodies To Citrullinated Alpha-Enolase Peptide 1 Are Specific For Rheumatoid Arthritis And Cross-React With Bacterial Enolase |
| 6 | 10.1080/03009742.2020.1795244 | Antibodies To Malondialdehyde-Acetaldehyde Modified Low-Density Lipoprotein In Patients With Newly Diagnosed Inflammatory Joint Disease |
| 7 | 10.3899/jrheum.091323 | Antibodies To Porphyromonas Gingivalis Are Associated With Anticitrullinated Protein Antibodies In Patients With Rheumatoid Arthritis And Their Relatives |
| 8 | 10.3390/jcm11041008 | Antibodies To Porphyromonas Gingivalis Are Increased In Patients With Severe Periodontitis, And Associate With Presence Of Specific Autoantibodies And Myocardial Infarction |
| 9 | 10.1136/annrheumdis-2021-eular.1580 | Antibodies To Porphyromonas Gingivalis Associate With The Presence Of Rheumatoid Arthritis-Related Autoantibodies In Patients With Periodontitis |
| 10 | 10.1136/annrheumdis-2014-eular.5077 | Antibodies To Porphyromonas Gingivalis In Patients With Rheumatoid Arthritis |
| 11 | 10.1002/art.39491 | Antibodies To Porphyromonas Gingivalis Indicate Interaction Between Oral Infection, Smoking, And Risk Genes In Rheumatoid Arthritis Etiology |
| 12 | 10.1016/j.anaerobe.2020.102230 | Porphyromonas : A Neglected Potential Key Genus In Human Microbiomes |
| 13 | 10.1902/jop.2011.110020 | Antibody Responses To Periodontopathic Bacteria In Relation To Rheumatoid Arthritis In Japanese Adults |
| 14 | 10.1016/j.intimp.2008.09.008 | Antibody Responses To Porphyromonas Gingivalis (P. Gingivalis) In Subjects With Rheumatoid Arthritis And Periodontitis |
| 15 | 10.3390/jcm8091309 | Rheumatoid Arthritis-Associated Mechanisms Of Porphyromonas Gingivalis And Aggregatibacter Actinomycetemcomitans |
| 16 | 10.1111/1756-185x.13977 | Anti-Carbamylated Protein And Peptide Antibodies As Potential Inflammatory Joint Biomarkers In The Relatives Of Rheumatoid Arthritis Patients |
| 17 | 10.1002/art.37911 | Anti-Citrullinated Protein Antibodies In Unaffected First-Degree Relatives Of Rheumatoid Arthritis Patients |
| 18 | 10.1016/j.peptides.2016.01.005 | Application Of Synthetic Peptides For Detection Of Anti-Citrullinated Peptide Antibodies |
| 19 | 10.1155/2019/2907062 | Are There Any Common Genetic Risk Markers For Rheumatoid Arthritis And Periodontal Diseases? A Case-Control Study |
| 20 | 10.1093/rheumatology/keaa219 | Arthritis Autoantibodies In Individuals Without Rheumatoid Arthritis: Follow-Up Data From A Dutch Population-Based Cohort (Lifelines) |
| 21 | 10.1902/jop.2013.120696 | Assessment Of Interleukin-6 Receptor Inhibition Therapy On Periodontal Condition In Patients With Rheumatoid Arthritis And Chronic Periodontitis |
| 22 | 10.1136/annrheumdis-2012-201593 | Association Between A History Of Periodontitis And The Risk Of Rheumatoid Arthritis: A Nationwide, Population-Based, Case-Control Study |
| 23 | 10.1007/s00393-017-0328-y | Association Between Anti-Porphyromonas Gingivalis Antibody, Anti-Citrullinated Protein Antibodies, And Rheumatoid Arthritis : A Meta-Analysis |
| 24 | 10.1186/s13075-020-2121-6 | Association Between Periodontitis And Anti-Citrullinated Protein Antibodies In Rheumatoid Arthritis Patients: A Cross-Sectional Study |
| 25 | 10.1002/art.39724 | Association Between Serum Antibodies To Periodontal Bacteria And Rheumatoid Factor In The Third National Health And Nutrition Examination Survey |
| 26 | 10.1016/j.identj.2020.12.015 | Association Between The Extent Of Periodontal Inflammation And The Severity Of Rheumatoid Arthritis In Japanese Patients With Rheumatoid Arthritis |
| 27 | 10.1111/1756-185x.13724 | Association Of Adipokines With Rheumatic Disease Activity Indexes And Periodontal Disease In Patients With Early Rheumatoid Arthritis And Their First-Degree Relatives |
| 28 | 10.1002/art.39118 | Association Of Anti-Porphyromonas Gingivalis Antibody Titers With Nonsmoking Status In Early Rheumatoid Arthritis: Results From The Prospective French Cohort Of Patients With Early Rheumatoid Arthritis |
| 29 | 10.3390/cimb43030103 | Association Of Bitter Taste Receptor T2R38 Polymorphisms, Oral Microbiota, And Rheumatoid Arthritis |
| 30 | 10.1002/jper.17-0616 | Association Of Different Immunosuppressive Medications With Periodontal Condition In Patients With Rheumatoid Arthritis: Results From A Cross-Sectional Study |
| 31 | 10.1002/art.40227 | Association Of Distinct Fine Specificities Of Anti-Citrullinated Peptide Antibodies With Elevated Immune Responses To Prevotella Intermedia In A Subgroup Of Patients With Rheumatoid Arthritis And Periodontitis |
| 32 | 10.1080/07853890.2020.1724321 | Association Of Rheumatoid Arthritis Disease Activity And Antibodies To Periodontal Bacteria With Serum Lipoprotein Profile In Drug Naive Patients |
| 33 | 10.1002/art.41572 | Associations Of Antibodies Targeting Periodontal Pathogens With Subclinical Coronary, Carotid, And Peripheral Arterial Atherosclerosis In Rheumatoid Arthritis |
| 34 | 10.1177/2380084419833694 | Attitudes Towards Oral Health In Patients With Rheumatoid Arthritis: A Qualitative Study Nested Within A Randomized Controlled Trial |
| 35 | 10.1111/jcpe.12727 | Autoantibodies Against Citrullinated Histone H3 In Rheumatoid Arthritis And Periodontitis Patients |
| 36 | 10.1136/annrheumdis-2019-216919 | Bacterial Citrullinated Epitopes Generated By Porphyromonas Gingivalis Infection-A Missing Link For ACPA Production |
| 37 | 10.1371/journal.pone.0192365 | Circulating Levels Of Carbamylated Protein And Neutrophil Extracellular Traps Are Associated With Periodontitis Severity In Patients With Rheumatoid Arthritis: A Pilot Case-Control Study |
| 38 | 10.1136/annrheumdis-2020-217589 | Circulating Microbial Small Rnas Are Altered In Patients With Rheumatoid Arthritis |
| 39 | 10.1080/20002297.2018.1487742 | Citrullination As A Plausible Link To Periodontitis, Rheumatoid Arthritis, Atherosclerosis And Alzheimer's Disease |
| 40 | 10.1016/j.archoralbio.2020.104695 | Citrullination In Periodontium Is Associated With Porphyromonas Gingivalis |
| 41 | 10.1007/s00784-015-1556-7 | Citrullination In The Periodontium-A Possible Link Between Periodontitis And Rheumatoid Arthritis |
| 42 | 10.1007/s10266-020-00566-0 | Clinical And Microbiological Effects Of Non-Surgical Periodontal Treatment In Individuals With Rheumatoid Arthritis: A Controlled Clinical Trial |
| 43 | 10.1186/ar4289 | Clinical Correlations With Porphyromonas Gingivalis Antibody Responses In Patients With Early Rheumatoid Arthritis |
| 44 | 10.1902/jop.2011.100481 | Clinical Periodontal And Microbiologic Parameters In Patients With Rheumatoid Arthritis |
| 45 | 10.1186/s13075-016-1100-4 | Concentration Of Antibodies Against Porphyromonas Gingivalis Is Increased Before The Onset Of Symptoms Of Rheumatoid Arthritis |
| 46 | 10.1136/annrheumdis-2016-eular.2147 | Concentrations Of Antibodies Against Porphyromonas Gingivalis Are Increased Before The Onset Of Symptoms Of Rheumatoid Arthritis |
| 47 | 10.1902/jop.2016.160422 | Correlation Of Periodontal Disease With Inflammatory Arthritis In The Time Before Modern Medical Intervention |
| 48 | 10.1902/jop.2016.160355 | Cross-Sectional Evaluation Of Periodontal Status And Microbiologic And Rheumatoid Parameters In A Large Cohort Of Patients With Rheumatoid Arthritis |
| 49 | 10.1177/1759720X19883152 | A Cross-Sectional Investigation Into The Association Between Porphyromonas Gingivalis And Autoantibodies To Citrullinated Proteins In A German Population |
| 50 | 10.1136/annrheumdis-2014-205385 | Defining The Role Of Porphyromonas Gingivalis Peptidylarginine Deiminase (PPAD) In Rheumatoid Arthritis Through The Study Of PPAD Biology |
| 51 | 10.1111/jcpe.12102 | Detection Of Oral Bacterial DNA In Synovial Fluid |
| 52 | 10.1111/j.1600-051X.2009.01496.x | Detection Of Periodontal Bacterial DNA In Serum And Synovial Fluid In Refractory Rheumatoid Arthritis Patients |
| 53 | 10.1002/acr.23411 | Disclosure Of Personalized Rheumatoid Arthritis Risk Using Genetics, Biomarkers, And Lifestyle Factors To Motivate Health Behavior Improvements: A Randomized Controlled Trial |
| 54 | 10.1080/00016357.2018.1469788 | Distribution Of Porphyromonas Gingivalis Fima Genotypes In Patients Affected By Rheumatoid Arthritis And Periodontitis |
| 55 | 10.1590/pboci.2020.107 | Diversity Of Sub-Gingival Fluids Microbiota Compositions In Periodontitis And Rheumatoid Arthritis Patients: A Case-Control Study |
| 56 | 10.1016/j.cellimm.2018.10.005 | Role Of The Intestinal Microbiome In Autoimmune Diseases And Its Use In Treatments |
| 57 | 10.1136/annrheumdis-2020-216972 | Dysbiosis In The Oral Microbiomes Of Anti-CCP Positive Individuals At Risk Of Developing Rheumatoid Arthritis |
| 58 | 10.1002/art.40485 | Dysbiotic Subgingival Microbial Communities In Periodontally Healthy Patients With Rheumatoid Arthritis |
| 59 | 10.3390/ijerph18052529 | Effect Of Anti-Rheumatic Treatment On The Periodontal Condition Of Rheumatoid Arthritis Patients |
| 60 | 10.1016/j.jbspin.2019.02.006 | The Effect Of Periodontal Treatment On Patients With Rheumatoid Arthritis: The ESPERA Randomised Controlled Trial |
| 61 | 10.1902/jop.2011.110420 | The Effects Of Chronic Periodontitis And Rheumatoid Arthritis On Serum And Gingival Crevicular Fluid Total Antioxidant/Oxidant Status And Oxidative Stress Index |
| 62 | 10.25100/cm.v52i3.5051 | Effects Of Nonsurgical Periodontal Therapy In Patients With Rheumatoid Arthritis: A Prospective Before And After Study |
| 63 | 10.1007/s00784-018-2420-3 | Effects Of Non-Surgical Periodontal Therapy On Periodontal Laboratory And Clinical Data As Well As On Disease Activity In Patients With Rheumatoid Arthritis |
| 64 | 10.1016/j.jbspin.2019.12.003 | Efficacy Of Baricitinib On Periodontal Inflammation In Patients With Rheumatoid Arthritis |
| 65 | 10.1038/s41598-021-82335-9 | Elevated Serum TREM-1 Is Associated With Periodontitis And Disease Activity In Rheumatoid Arthritis |
| 66 | 10.1016/j.jbspin.2010.04.015 | Rheumatoid Arthritis And Periodontal Disease |
| 67 | 10.1007/s11926-011-0203-9 | Environmental Exposures And Rheumatoid Arthritis Risk |
| 68 | 10.7860/jcdr/2017/24412.9792 | Estimation Of Pentraxin 3 And Porphyromonas Gingivalis Levels In Patients With Rheumatoid Arthritis And Periodontitis- An Observational Study |
| 69 | 10.1111/jre.12288 | Expression Of Anti-Porphyromonas Gingivalis Peptidylarginine Deiminase Immunoglobulin G And Peptidylarginine Deiminase-4 In Patients With Rheumatoid Arthritis And Periodontitis |
| 70 | 10.1111/jre.12288 | Expression Of Anti-Porphyromonas Gingivalis Peptidylarginine Deiminase Immunoglobulin G And Peptidylarginine Deiminase-4 In Patients With Rheumatoid Arthritis And Periodontitis |
| 71 | 10.1016/j.archoralbio.2018.10.022 | Expression Of Human And Porphyromonas Gingivalis Glutaminyl Cyclases In Periodontitis And Rheumatoid Arthritis-A Pilot Study |
| 72 | 10.4067/s0034-98872015001200006 | Frequency And Severity Of Periodontitis Among Patients With Rheumatoid Arthritis |
| 73 | 10.3389/fimmu.2016.00080 | Hand To Mouth: A Systematic Review And Meta-Analysis Of The Association Between Rheumatoid Arthritis And Periodontitis |
| 74 | 10.1136/annrheumdis-2012-202726 | Heightened Immune Response To Autocitrullinated Porphyromonas Gingivalis Peptidylarginine Deiminase: A Potential Mechanism For Breaching Immunologic Tolerance In Rheumatoid Arthritis |
| 75 | 10.3390/jcm10215153 | Humoral Response To Microbial Biomarkers In Rheumatoid Arthritis Patients |
| 76 | 10.1136/annrheumdis-2015-208495 | Identification Of An Immunodominant Peptide From Citrullinated Tenascin-C As A Major Target For Autoantibodies In Rheumatoid Arthritis |
| 77 | 10.1128/cdli.10.6.1043-1050.2003 | Immunoglobulin G And A Antibody Responses To Bacteroides Forsythus And Prevotella Intennedia In Sera And Synovial Fluids Of Arthritis Patients |
| 78 | 10.1016/j.pdpdt.2021.102698 | Impact Of Photodynamic Therapy As An Adjunct To Non-Surgical Periodontal Treatment On Clinical And Biochemical Parameters Among Patients Having Mild Rheumatoid Arthritis With Periodontitis |
| 79 | 10.1002/art.39514 | Impaired Porphyromonas Gingivalis-Induced Tumor Necrosis Factor Production By Dendritic Cells Typifies Patients With Rheumatoid Arthritis |
| 80 | 10.1016/j.clim.2014.02.011 | In Black Africans With Rheumatoid Arthritis, ACPA Recognize Citrullinated Fibrinogen And The Derived Peptides Α36-50Cit38,42 And Β60-74Cit60,72,74, Like In Caucasians |
| 81 | 10.1007/s00296-017-3779-1 | Incidence Of Inflammatory Joint Diseases In Finland: Results From A Population-Based Epidemiological Study |
| 82 | 10.3390/jcm8050751 | Infliximab Induced A Dissociated Response Of Severe Periodontal Biomarkers In Rheumatoid Arthritis Patients |
| 83 | 10.1002/jper.17-0575 | Influence Of Plaque Control On The Relationship Between Rheumatoid Arthritis And Periodontal Health Status Among Japanese Rheumatoid Arthritis Patients |
| 84 | 10.1111/odi.14147 | Inhibition Of Rgs10 Aggravates Periodontitis With Collagen-Induced Arthritis Via The Nuclear Factor-Kappab Pathway |
| 85 | 10.2334/josnusd1959.37.197 | Levels Of Serum Igg Against Porphyromonas Gingivalis In Patients With Rapidly Progressive Periodontitis, Rheumatoid Arthritis And Adult Periodontitis |
| 86 | 10.1172/jci.insight.90045 | A Molecular Signature Of Preclinical Rheumatoid Arthritis Triggered By Dysregulated PTPN22 |
| 87 | 10.3390/ijerph18189560 | No Obvious Role For Suspicious Oral Pathogens In Arthritis Development |
| 88 | 10.1002/JPER.20-0295 | Novel And Known Periodontal Pathogens Residing In Gingival Crevicular Fluid Are Associated With Rheumatoid Arthritis |
| 89 | 10.1155/2022/6839356 | Oral And Intestinal Bacterial Substances Associated With Disease Activities In Patients With Rheumatoid Arthritis: A Cross-Sectional Clinical Study |
| 90 | 10.1186/s12969-019-0387-5 | Oral Health And Plaque Microbial Profile In Juvenile Idiopathic Arthritis |
| 91 | 10.1016/S0973-3698(11)60072-1 | Oral Hygiene And Periodontal Status In A Group Of Patients With Rheumatoid Arthritis |
| 92 | 10.1136/annrheumdis-2017-eular.5034 | Oral Microbiome Profile In Rheumatoid Arthritis Patients: Association Between Tongue Biofilm Porphyromonas Gingivalis Amount And Disease Activity |
| 93 | 10.3389/fcimb.2019.00475 | Oral Microbiota Perturbations Are Linked To High Risk For Rheumatoid Arthritis |
| 94 | 10.1002/art.30186 | Particular Association Of Clinical And Genetic Features With Autoimmunity To Citrullinated Alpha-Enolase In Rheumatoid Arthritis |
| 95 | 10.1136/annrheumdis-2011-201232.3 | Patients With Rheumatoid Arthritis And Periodontitis Have Higher Disease Activity And A More Pronounced Antibody Response Against Porphyromonas Gingivalis |
| 96 | 10.3390/microorganisms8060869 | Porphyromonas Gingivalis, A Long-Range Pathogen: Systemic Impact And Therapeutic Implications |
| 97 | 10.7860/jcdr/2018/36791.12158 | Periodontal Conditions During Arthritis Therapy With TNF-Alpha Blockers |
| 98 | 10.1007/s00296-019-04460-z | Periodontal Disease And Influence Of Periodontal Treatment On Disease Activity In Patients With Rheumatoid Arthritis And Spondyloarthritis |
| 99 | 10.1016/j.berh.2017.08.001 | Periodontal Disease And Periodontal Bacteria As Triggers For Rheumatoid Arthritis |
| 100 | 10.1002/art.34539 | Periodontal Disease And The Oral Microbiota In New-Onset Rheumatoid Arthritis |
| 101 | 10.1902/jop.2015.150455 | Periodontal Disease In Individuals With A Genetic Risk Of Developing Arthritis And Early Rheumatoid Arthritis: A Cross-Sectional Study |
| 102 | 10.1111/j.1600-051X.2011.01776.x | Periodontal Disease, Tooth Loss And Incident Rheumatoid Arthritis: Results From The First National Health And Nutrition Examination Survey And Its Epidemiological Follow-Up Study |
| 103 | 10.3390/jcm8050630 | Periodontal Health And Oral Microbiota In Patients With Rheumatoid Arthritis |
| 104 | 10.1002/JPER.20-0051 | Periodontal Inflammation And Distinct Inflammatory Profiles In Saliva And Gingival Crevicular Fluid Compared With Serum And Joints In Rheumatoid Arthritis Patients |
| 105 | 10.5051/jpis.2018.48.6.347 | Periodontal Pathogens And The Association Between Periodontitis And Rheumatoid Arthritis In Korean Adults |
| 106 | 10.1093/rheumatology/kev274 | Periodontal Pathogens Participate In Synovitis In Patients With Rheumatoid Arthritis In Clinical Remission: A Retrospective Case-Control Study |
| 107 | 10.1902/jop.2013.130079 | Periodontal Treatment Decreases Levels Of Antibodies To Porphyromonas Gingivalis And Citrulline In Patients With Rheumatoid Arthritis And Periodontitis |
| 108 | 10.1002/art.38348 | Periodontitis And Porphyromonas Gingivalis In Patients With Rheumatoid Arthritis |
| 109 | 10.1371/journal.pone.0122121 | Periodontitis And Porphyromonas Gingivalis In Preclinical Stage Of Arthritis Patients |
| 110 | 10.1016/j.rbre.2014.03.007 | Periodontitis Exposure Within One Year Before Anti-Diabetic Treatment And The Risk Of Rheumatoid Arthritis In Diabetes Mellitus Patients: A Population-Based Cohort Study |
| 111 | 10.1136/bmjopen-2016-011916 | Periodontitis In Early And Chronic Rheumatoid Arthritis: A Prospective Follow-Up Study In Finnish Population |
| 112 | 10.1186/ar4061 | Periodontitis In Established Rheumatoid Arthritis Patients: A Cross-Sectional Clinical, Microbiological And Serological Study |
| 113 | 10.3904/kjim.2015.202 | Periodontitis Is Associated With Rheumatoid Arthritis: A Study With Longstanding Rheumatoid Arthritis Patients In Korea |
| 114 | 10.1111/jcpe.12485 | Periodontitis Prevalence And Serum Antibody Reactivity To Periodontal Bacteria In Primary Sjogren's Syndrome: A Pilot Study |
| 115 | 10.1016/j.cct.2014.08.007 | Personalized Risk Estimator For Rheumatoid Arthritis (PRE-RA) Family Study: Rationale And Design For A Randomized Controlled Trial Evaluating Rheumatoid Arthritis Risk Education To First-Degree Relatives |
| 116 | 10.1002/art.34595 | Porphyromonas Gingivalis And Disease-Related Autoantibodies In Individuals At Increased Risk Of Rheumatoid Arthritis |
| 117 | 10.1186/ar4243 | Porphyromonas Gingivalis And The Pathogenesis Of Rheumatoid Arthritis: Analysis Of Various Compartments Including The Synovial Tissue |
| 118 | 10.1111/cei.13184 | Porphyromonas Gingivalis In The Tongue Biofilm Is Associated With Clinical Outcome In Rheumatoid Arthritis Patients |
| 119 | 10.11607/prd.2656 | A Possible Link Between Rheumatoid Arthritis And Periodontitis: A Systematic Review And Meta-Analysis |
| 120 | 10.4103/jfmpc.jfmpc_398_20 | Prevalence And Severity Of Periodontitis In Patients With Established Rheumatoid Arthritis And Osteoarthritis |
| 121 | 10.1001/jamanetworkopen.2019.5394 | Prevalence Of Periodontal Disease And Periodontopathic Bacteria In Anti-Cyclic Citrullinated Protein Antibody-Positive At-Risk Adults Without Arthritis |
| 122 | 10.1016/j.jim.2014.03.013 | Preventing Intense False Positive And Negative Reactions Attributed To The Principle,Of ELISA To Re-Investigate Antibody Studies In Autoimmune Diseases |
| 123 | 10.1080/00016357.2021.1959053 | Relation Between Anti-Porphyromonas Gingivalis Antibody Titers And HLA-DRB1 Neutral Alleles In Individuals With Rheumatoid Arthritis |
| 124 | 10.1136/annrheumdis-2019-215962 | Response To: 'Response To: 'Alcohol Is Not The Missing Link Between Porphyromonas Gingivalis Related Periodontitis And Radiologic Progression In Early Rheumatoid Arthritis' By Hillion Et Al' By Marotte And Paul |
| 125 | 10.1007/s10389-019-01073-5 | Rheumatoid Arthritis And Periodontitis: A Jordanian Case-Control Study |
| 126 | 10.3904/kjim.2018.093 | Rheumatoid Arthritis Is Associated With Early Tooth Loss: Results From Korea National Health And Nutrition Examination Survey V To VI |
| 127 | 10.1111/1756-185x.13590 | Rheumatoid Arthritis Patients' Oral Health And Disease Activity |
| 128 | 10.1016/j.jbspin.2020.04.024 | Rheumatoid Arthritis Risk In Periodontitis Patients: A Systematic Review And Meta-Analysis |
| 129 | 10.1186/s13075-015-0690-6 | Rheumatoid Arthritis-Associated Autoantibodies In Non-Rheumatoid Arthritis Patients With Mucosal Inflammation: A Case-Control Study |
| 130 | 10.5152/eurjrheum.2018.17111 | Risk Factor Assessment Of Rheumatoid Arthritis In North Kerala |
| 131 | 10.1177/039463201002300234 | The Role Of Anti-Cyclic Citrullinated Peptide Antibody In Periodontal Disease |
| 132 | 10.1093/rheumatology/kez368 | Role Of Good Oral Hygiene On Clinical Evolution Of Rheumatoid Arthritis: A Randomized Study Nested In The ESPOIR Cohort |
| 133 | 10.1002/cre2.68 | Salivary Ammonia Levels And Tannerella Forsythia Are Associated With Rheumatoid Arthritis: A Cross Sectional Study |
| 134 | 10.1016/j.imbio.2012.04.011 | Salivary Iga Antibodies To Cyclic Citrullinated Peptides (CCP) In Rheumatoid Arthritis |
| 135 | PMPMID: **16369381** | Serum Antibodies To Oral Anaerobic Bacteria In Patients With Rheumatoid Arthritis |
| 136 | 10.1007/s00784-016-1938-5 | Serum Antibody Levels Against Porphyromonas Gingivalis In Patients With And Without Rheumatoid Arthritis - A Systematic Review And Meta-Analysis |
| 137 | 10.1016/j.jaut.2015.03.002 | Significant Association Of Periodontal Disease With Anti-Citrullinated Peptide Antibody In A Japanese Healthy Population - The Nagahama Study |
| 138 | 10.1016/j.jaut.2015.03.002 | Significant Association Of Periodontal Disease With Anti-Citrullinated Peptide Antibody In A Japanese Healthy Population – The Nagahama Study |
| 139 | 10.1186/s12891-015-0792-y | Smoking, Porphyromonas Gingivalis And The Immune Response To Citrullinated Autoantigens Before The Clinical Onset Of Rheumatoid Arthritis In A Southern European Nested Case-Control Study |
| 140 | 10.1016/j.annepidem.2019.03.006 | Sociodemographic Variation In The Oral Microbiome |
| 141 | 10.1016/j.jobcr.2021.02.010 | Soluble Neuropilin-1 In Gingival Crevicular Fluid Is Associated With Rheumatoid Arthritis: An Exploratory Case-Control Study |
| 142 | 10.1007/s00005-022-00649-6 | Study Of Plasma Anti-CD26 Autoantibody Levels In A Cohort Of Treatment-Naive Early Arthritis Patients |
| 143 | WOS:000410388000056 | Study On The Prevalence Of Periodontopathogenic Bacteria In Serum And Subgingival Bacterial Plaque In Patients With Rheumatoid Arthritis |
| 144 | 10.1093/rheumatology/key052 | The Subgingival Microbiome In Patients With Established Rheumatoid Arthritis |
| 145 | 10.1186/s12903-021-01597-x | Subgingival Microbiome Of Deep And Shallow Periodontal Sites In Patients With Rheumatoid Arthritis: A Pilot Study |
| 146 | 10.1371/journal.pone.0202278 | Subgingival Microbiome Of Rheumatoid Arthritis Patients In Relation To Their Disease Status And Periodontal Health |
| 147 | 10.3390/pathogens10020193 | The Subgingival Plaque Microbiome, Systemic Antibodies Against Bacteria And Citrullinated Proteins Following Periodontal Therapy |
| 148 | 10.1186/s12969-016-0068-6 | Symptoms Of Periodontitis And Antibody Responses To Porphyromonas Gingivalis In Juvenile Idiopathic Arthritis |
| 149 | 10.1111/1756-185X.13240 | Rheumatoid Arthritis And Periodontal Disease: What Are The Similarities And Differences? |
| 150 | 10.1111/jre.12299 | TLR4 Asp299Gly Polymorphism May Be Protective Against Chronic Periodontitis |
| 151 | 10.1111/jre.12314 | Tumor Necrosis Factor-Alpha Gene Promoter Methylation In Japanese Adults With Chronic Periodontitis And Rheumatoid Arthritis |
| 152 | 10.1016/j.cyto.2020.155117 | Upregulation Of Circulating Inflammatory Biomarkers Under The Influence Of Periodontal Disease In Rheumatoid Arthritis Patients |
| 153 | 10.1007/s11926-012-0314-y | The Role Of The Microbiome In Rheumatic Diseases |
| 154 | 10.1111/1756-185x.12428 | Rheumatic Diseases And The Microbiome |
| 155 | 10.1007/s10165-009-0194-9 | Rheumatoid Arthritis Is Linked To Oral Bacteria: Etiological Association |
| 156 | 10.3390/pathogens9110944 | Porphyromonas Gingivalis And Its Systemic Impact: Current Status |
